# Improving pharmacy practice in relation to complementary medicines: a qualitative study evaluating the acceptability and feasibility of a new ethical framework in Australia

**DOI:** 10.1186/s12910-020-00570-7

**Published:** 2021-01-06

**Authors:** Amber Salman Popattia, Laetitia Hattingh, Adam La Caze

**Affiliations:** 1 School of Pharmacy, The University of Queensland, St. Lucia, 4072 Australia; 2 Gold Coast Hospital and Health Service, Southport, Australia; 3 School of Pharmacy and Pharmacology, Griffith University, Gold Coast, Australia

**Keywords:** Pharmacy ethics, Professional ethics, Pharmacy practice, Complementary medicines, Responsibilities

## Abstract

**Background:**

There is a need for clearer guidance for pharmacists regarding their responsibilities when selling complementary medicines. A recently published ethical framework provides guidance regarding the specific responsibilities that pharmacists need to meet in order to fulfil their professional obligations and make a positive contribution to health outcomes when selling complementary medicines.

**Objective:**

Evaluate the acceptability and feasibility of a new ethical framework for the sale of complementary medicines in community pharmacy.

**Methods:**

Australian community pharmacists were invited to participate in online focus groups and interviews. Participants were recruited via multiple methods, including social media and the professional networks of pharmacy groups. Participants were provided the ethical framework prior to the discussion. Discussions were transcribed verbatim and analysed using thematic analysis.

**Results:**

Seventeen community pharmacists participated in the study (11 in 4 focus groups and 6 in individual interviews). There was good representation among participants in terms of gender, years of practice, pharmacy location and script volume. Participants differed in how proactive they were in relation to selling and providing advice on complementary medicines, how they interpreted evidence in relation to complementary medicines, and how they navigated their practice within the retail environment of community pharmacy. The majority of participants found the framework was acceptable for practice and was feasible for implementation with targeted support. Participants identified two important areas for targeted support in implementing the framework: improved access to evidence-based information resources on complementary medicines and independent evidence-based education and training on complementary medicine for pharmacists and pharmacy support staff.

**Conclusion:**

The ethical framework addresses an important gap in providing specific professional guidance to pharmacists when selling complementary medicines. The results of the study suggest that the framework may be acceptable to community pharmacists and be feasible to implement with targeted support.

## Background

Complementary medicines are a $4.9 billion dollar industry in Australia, 41% of which is sold through pharmacies [1]. Complementary medicines include products that contain herbs, vitamins, nutritional supplements, homeopathic treatments and aromatherapy preparations. While the terms used to refer to these products vary internationally, there are many similarities in regulation and use of complementary medicines in Australasia, Europe and North America. Consumers purchase complementary medicines from pharmacies due to a trust in the quality of the products and the availability of advice [2–4]. However, recent media reports in Australia suggest that pharmacists are failing to meet community expectations in relation to the sale of complementary medicines [5–7]. While there is clear recognition from professional bodies that pharmacists should (1) support consumer choice (2) provide advice that is informed by evidence, and (3) seek to prevent harm related to complementary medicines [8–11], there is limited guidance on what to do in response to the conflict between these general principles. Despite many consumers choosing to use complementary medicines, many of these products lack rigorous evidence of effectiveness, and, despite relative safety, can cause harm through adverse effects, drug interactions and delayed treatment [12, 13]. Conflicts arise for pharmacists between respecting the autonomy of consumers who choose to use complementary medicines and professional responsibilities regarding evidence-based practice and supporting positive health outcomes. This conflict is exacerbated by the competitive retail environment of community pharmacy.

There are 5762 community pharmacies in Australia, located throughout urban, region and rural areas [14]. Community pharmacies in Australia dispense medicines subsidized by the national Pharmaceutical Benefits Scheme, provide health advice, manage minor ailments and provide additional services such as vaccination and blood pressure monitoring. Most pharmacies have a retail component, which includes cosmetics, health products for minor conditions and complementary medicines. Pharmacies are either independently operated, or operated as part of one of several banner groups. Banner groups support various business and marketing operations within the pharmacy. Pharmacies are located in shopping centres, among local businesses in ‘shopping strips’, or co-located with medical centres. ‘Discount pharmacies’ have been a successful and increasing service model over the past 10–15 years. These pharmacies are often warehouse-style pharmacies with a significant retail component (including complementary medicines) and compete on the basis of low price.

The most common degree format for Australian pharmacists is a 4 year Bachelor program with one year of supervised practice before registration as a pharmacist. Pharmacy school curricula need to meet accreditation standards and produce graduates that are able to meet defined competency standards [15]. The need for further training on evidence-based complementary medicines has been identified [16]. Pharmacy support staff are employed to assist consumers and often play an important role in assisting consumers with requests for complementary medicines [17]. Pharmacy support staff undergo vocational training, this training includes general product and health information to assist consumers as well as identifying the types of queries that need to be referred to the pharmacist. Some pharmacies employ naturopaths to consult with consumers in relation to complementary medicines. Naturopaths in Australia typically complete either a Bachelor degree or Advanced Diploma in natural therapies and may be registered with one of a number of professional associations [18].

Recent work has sought to identify the responsibilities of pharmacists in relation to complementary medicines discussed in the literature [16, 19–22]. Key responsibilities include acknowledging and documenting use, being knowledgeable about complementary medicines, ensuring safe use, reporting adverse drug reactions, educating consumers about use and collaborating with health professionals [19]. A lack of clarity regarding the roles and ethical responsibilities of pharmacists in relation to complementary medicines has been identified as a key barrier to improving practice [21–23]. To the extent that ethical considerations are discussed in the pharmacy literature, the conflict between supporting consumer choice and respecting autonomy, evidence-based practice and business considerations are frequently identified but rarely addressed. Part of the problem is the lack of explicit ethical principles to guide decision-making.

In response to these gaps, an ethical framework has been developed by two of the authors of this paper (ASP and AL) that provides specific guidance to pharmacists regarding their responsibilities when selling complementary medicines [24]. The framework seeks to identify the responsibilities that pharmacists need to meet in order to ensure that the sale of complementary medicines in community pharmacy promotes good consumer health outcomes. There are three components to the framework: principle-based ethics provides the theoretical foundations, a *public health argument* provides support for the sale of complementary medicines in community pharmacy, and specific responsibilities are provided that ensure that pharmacists meet their obligations to consumers when selling complementary medicines. An overview of the framework is provided below, a more detailed argument can be found in [24].

The framework employs the ‘four principles approach’ to bioethics provided by Beauchamp and Childress in *Principles of Bioethics* [25]. Richardson’s account of specification within the four principles approach is used to (1) identify the links between one or more of the four principles of bioethics and widely accepted professional practice norms that relate to how pharmacists approach the sale of complementary medicines and (2) resolve conflicts in the practice norms in relation to the sale of complementary medicines by further specifying the practice norm to address the conflict [26, 27]. Key professional practice norms include the need for pharmacists to respect consumer health preferences; to support the informed choice of consumers in relation to treatment decisions; to provide advice that supports positive health outcomes and avoids harms, and to provide evidence-based care. These practice norms are specifications of the more general principles of respect for autonomy, beneficience and non-maleficience, and can be further specified to provide the explicit advice provided within the responsibilities outlined in the framework [24].

Most discussion surrounding whether or not pharmacists should sell complementary medicines focuses on the lack of sufficiently rigorous evidence to support the effectiveness of many of these products. Views on this topic tends to fall into one of two camps: the view that pharmacists should not sell complementary medicines due to the lack of evidence of effectiveness (in this setting any harm, no matter how mild or unlikely, will be enough to trump the lack of evidence of benefit), and the view that it is appropriate for pharmacists to sell complementary medicines that lack evidence of effectiveness based on consumer preference (see, for example, the discussion in [5]). Each of these views has limitations. The view that pharmacists should not be involved in the sale of complementary medicines insufficiently acknowledges (1) that in most countries complementary medicines are regulated as safe for self-care, are widely available from a range of outlets, and are likely to remain widely used even if removed from pharmacies, and (2) the contribution that pharmacists can make in supporting wise choices in relation to using complementary medicines, especially in relation to avoiding drug interactions and other harms. Conversely, the view that pharmacists should sell complementary medicines based on consumer preference neglects the commitments that pharmacists have to evidence-based practice and supporting informed choice. The public health argument seeks to address these limitations.

The public health argument proposes that it is appropriate for pharmacists to sell complementary medicines in community pharmacy providing the public benefits outweigh the harms, and the actions of the pharmacist are consistent with professional norms. The public health argument is an attempt to break the impasse between those that favour a strict evidence-based approach and those that favour a more permissive approach based on consumer preference. It does this by shifting the primary locus of justification for pharmacists selling complementary medicines from an assessment of benefits and risks at an individual level to a community level. This shift facilitates the recognition that pharmacists are accessible health professionals with the training and skills to provide guidance on the appropriate use of complementary medicines. Complementary medicines are regulated as being sufficiently safe for self-care and are likely to be widely used by consumers whether or not they are available in pharmacies. Having complementary medicines available in pharmacies provides an avenue for consumers to receive evidence-based advice on the efficacy and safety of these treatments. However, in order for the public to benefit from the sale of complementary medicines in pharmacies, pharmacists need to meet a number of responsibilities in relation to these products.

Table 1 provides the specific responsibilities the framework proposes that pharmacists should meet when selling and recommending complementary medicines and managing staff in relation to these products. The framework makes a distinction between pharmacy staff making an *explicit recommendation* to take a complementary medicine, and selling complementary medicines without an explicit recommendation. The framework suggests that *recommendations* for a complementary medicine must be consistent with current best evidence, and all *sales* of a complementary medicine should be accompanied with an *offer* of advice from a pharmacist. A pharmacist must be available to provide that advice and to provide sufficient information to the consumer such that they can make an informed decision with regard to the purchase of the complementary medicine. Since some consumers might refuse the offer of advice from a pharmacy, it is the responsibility of the pharmacist to have procedures in place to identify and intervene if a consumer is at significant risk of harm from complementary medicines.

**Table 1:** The key responsibilities of pharmacists when selling complementary medicines

Responsibilities	
1. Pharmacists should provide evidence-based recommendations to consumers regarding complementary medicines	
2. Pharmacists should train all staff in a pharmacy to ensure that they provide evidence-based recommendations regarding complementary medicines and refer to a pharmacist when required	
3. When providing advice, pharmacists should provide sufficient information for consumers to make informed decisions regarding complementary medicines	
4. Pharmacists should setup the pharmacy so that consumers are provided an offer of advice from a pharmacist when purchasing complementary medicines; pharmacists should be available to provide that advice	
5. Pharmacists must be vigilant for possible harms related to complementary medicines and intervene if risk of harm is significant	

The aim of this study was to evaluate the acceptability and feasibility of the proposed ethical framework for the sale of complementary medicines in community pharmacy.

## Methods

Australian community pharmacists were invited to participate in online workshops in September and October 2019. Pharmacists were recruited via multiple channels. Social media was used to advertise the study through professional organisations and professional groups. A number of national pharmacy banner groups advertised the study through internal email distribution lists. In addition, approximately 20 regional and rural pharmacies were also contacted directly to advertise the study in an attempt to ensure participation from pharmacists working outside of urban settings. Purposive sampling was employed to ensure that the age and gender distribution of participants reflected the workforce and that participants were recruited with different levels of experience and from different practice environments (small independent pharmacies, large chains, and discount-oriented pharmacies).

The workshops employed focus group methods to engage participants in discussion regarding the sale of complementary medicines in community pharmacies. Focus groups provide an opportunity to investigate complex behaviours and motivations, to learn more about the degree of consensus on a topic, and to gain feedback regarding new ideas [28, 29]. They are especially helpful to understand group norms, meanings and processes [30]. Workshops were offered inside and outside of usual business hours using video-conferencing software, Zoom. Conducting focus groups via video-conference provided an opportunity to recruit participants from a large geographical area. A number of strategies were employed to support the success of conducting the focus group in an online environment, these included seeking to arrange groups of 4–6 participants (limiting larger groups), enabling video feeds and offering alternatives for those with lower internet speeds [31]. Semi-structured interviews were conducted with participants who were unable to join a focus group. Participants were asked to review information about the framework prior to the focus group or interview (see Additional file 1). The discussion topics explored in the semi-structured interview were developed for the study (see Additional file 2). Discussion topics explored the context in which pharmacists provide advice on complementary medicines within community pharmacy, and the acceptability and feasibility of the proposed ethical framework. The semi-structured interviews followed the same structure as the focus groups.

It was made clear from the start of each focus group and interview that the objective of the discussion was to understand the participants’ views and how they varied. Participants were encouraged to share diverging views and to debate topics in a respectful manner. All focus groups and interviews were conducted by AL. The facilitator did not share views during the focus groups or interviews. ASP was an observer for most of the focus groups and interviews. AL and ASP debriefed immediately following each focus group and interview and prepared a summary that was sent to participants for comment.

The focus groups and interviews were video and audio recorded and then transcribed verbatim. The transcripts were analysed using the thematic analysis methods described by Braun and Clarke [32]. An inductive approach to coding was employed, and themes were developed with a focus on addressing the study research questions. Two investigators, AL and ASP, familiarized themselves with the data and developed an initial coding scheme. This was refined through discussion early in the analysis and then used to code the focus groups and interviews. AL and ASP then identified and refine themes individually first, and then as a group that included LH. Focus groups and interviews are labelled as “Discussions” and numbered in order. Participants, “P”, are also allocated a number in order. Whether the discussion was a focus group or interview is indicated by “F” or “I”. “D1P1-I” refers to Discussion 1, Participant 1, Interview.

All three investigators have experience in pharmacy ethics. AL has experience in qualitative research, facilitation of online groups and research and teaching in ethical reasoning and decision-making in pharmacy practice. ASP has a background in nursing and bioethics and developed the ethical framework with AL as part of her PhD. LH has experience in health law and ethics and qualitative research. LH was involved in development of the Pharmaceutical Society of Australia *Code of ethics for pharmacists*.

## Results

Seventeen community pharmacists participated in 4 workshops and 6 individual interviews. The workshops contained 2–4 participants and went from 29 to 68 min in duration (median duration 42.5 min). The duration of the interviews ranged from 17 to 34 min (median 21 min). Demographic features of the participants are provided in Table 2. Participants varied in terms of gender, years of practice and type of pharmacy and typical script volume. More than a third of participants worked in a regional or rural location.

**Table 2:** Participant demographics

Demographics		N
*Gender*		
Male		9
Female		8
*Years of practice*		
1–5		5
6–10		6
11–20		3
20+		3
*Type of pharmacy*		
Large shopping centre		5
Small local		6
Extended hours		3
Discount		3
*Location*		
Urban		11
Regional		5
Rural		1
*Script volume*		
0–50 prescriptions		1
50–100 prescriptions		4
100–200 prescriptions		1
200–300 prescriptions		3
300+ prescriptions		8

Participants self-selected the location and type of pharmacy. Locations were defined as follows: ‘Urban area’: major cities; ‘Regional area’: Large towns and cities not far from major cities; ‘Rural area’: small towns typically located further from major cities. Participants were able to select more than one descriptor of the type of pharmacy they worked in, but all selected only one descriptor

### Key themes

A number of themes were consistently discussed in the focus groups and interviews. Thematic saturation occurred after 3 focus groups and 4 interviews (13 participants), additional focus groups and interviews helped to explore and confirm key findings. Three main themes emerged from the focus groups and interviews. These themes are summarised in Table 3. The first two themes represent spectra on which participants differed: *Approach to complementary medicines (proactive–reactive)* and *Approach to evidence*. The third theme, *Navigating practice in a retail environment*, represents the recognition from all participants that community pharmacy is in a retail environment and decisions regarding professional practice have resource, work flow and other financial implications.

**Table 3:** Key themes from the focus groups and interviews and the relevance these themes had on the context of complementary medicine sales in community pharmacy and the perceived acceptability and feasibility of the proposed framework

Theme	Relevance
Approach to complementary medicines	Context, feasibility
Approach to evidence	Context, acceptability
Navigating practice in a retail environment	Acceptability, feasibility

The ways in which participants *approached complementary medicines*, *approached evidence*, and *navigated practice in a retail environment* inform how they viewed their responsibilities in relation to complementary medicines within the context of community pharmacy practice. How participants approached these key themes also informed their views on the acceptability and feasibility of the proposed framework. Each of these themes are briefly introduced below. Subsequent sections provide further discussion regarding how participant responses within these themes addresses the objectives of the project. Understanding these themes, and the ways in which participants varied within the themes provide insight into the *acceptability* and *feasibility* of the framework as perceived by the participants.

#### Approach to complementary medicines

A number of participants described their practice in terms of a proactive approach to complementary medicines. These participants tended to initiate discussion regarding complementary medicines with consumers and see an important role for pharmacists in being proactive in relation to complementary medicines (Table 4, quote 1). Some participants worked in community pharmacies with a specific set-up facilitating provision of specialist advice on complementary medicines, including the use of practitioner-only lines (Table 4, quote 2). ‘Practitioner-only’ lines are complementary medicines that are regulated in the same was as other complementary medicines, but are *marketed* such that they are only available from certain types of practitioners. Historically, these products have been sold by naturopaths, but in recent years pharmacists have become more actively involved in these sales.

**Table 4:** Quotes from participants supporting the key themes and the variation within key themes

	Participant quotes
	*Approach to complementary medicines*
1	Pharmacists are becoming more involved than before. People are trusting pharmacists more. They always check their complementary medicine. I think, from what I remember five years ago, people were just picking it up. They were thinking that, “That’s just a supplement,” but I think the awareness is more than before among people. So they always come and ask, “Oh, is this one safe?” or, “What should I take?” I think now, lots of pharmacists are always checking things for them. (D1P1-I)
2	I work in a small community pharmacy. I probably consider myself a integrated pharmacist. We have complementary medicines in three different areas similar to the rest of your medicines, like S2s, S3s [in Australia schedule 2 (S2) products are “pharmacy only” products and S3 products are “pharmacist only” products]. So we’ve got some out in the front shop, which I consider your [day-to-day] vitamins, like your supermarket lines. They’re more lines that are more for patients to choose and that sort of thing if they want to self-select. If they go for advice from a pharmacist or staff member, we’d probably go for something a bit better quality. So we’ve got some in the S2 section which are, I guess, better quality practitioner ranges. And then we’ve got your other ones in your S3 areas which are ranges that do require a consult or a prescription. So a lot of them are prescribed by some of our doctors as well. (D7P13-F)
3	I suppose it’s not as big a focus in my professional practice…. I think it’s probably because of lack of knowledge, to be honest, and confidence, where you feel a lot more secure at the back counter or in a dispensary than you do out in the vitamin section. (D5P8-F)
4	Yeah. I think at the moment, I don’t think we have much role to play in selling or providing any counselling for complementary medicine because first, working in community pharmacy, our main role is actually just dispensing. And then pharmacies, I think, we should follow more like evidence-based medicine practise…. All this supplementary of complementary medicine and all, they’re not evidence-based. (D5P6-F)
	*Approach to evidence*
5	[W]e shouldn’t just be selling things because someone… says, “Oh, this turmeric is great for the sake of curing cancer.” I think there has to be some level of evidence… And it’s hard in certain conditions because you’re just never going to have the trials. (D4P5-I)
6	If we, I think, perhaps as an industry, move towards more– well, what is the evidence? Do I feel that your needs will be met by what I’m recommending today? Is there evidence to support what it says on the label, or what it says in the marketing material? And if not, then maybe we, as an industry, could push the emphasis of companies bringing things to market, being more about actual evidence. More money going into these studies of N equals 50. (D5P9-F)
7	So how I would actually interpret [guidance to only sell complementary medicines that have evidence of effectiveness], and this is where placebo effects comes in. So hey, if it’s not doing them any harm and they think it’s better for them and they’re going on in their life and happy days, you just let them go. (D5P7-F)
8	So I see a lot of people really—a lot of people want to use it. I’ve talked to a lot of customers, and they do feel the result. Every time they come back, I always ask, “Is this working for you?” And a lot of times, they say, “Yes, I know it’s working because when my bottle ran out, I started feeling it.” So then they came back to get a new bottle. So regarding your first question where you say, “What’s our perspective regarding purchase of natural medications?” I think they really work. I think they work depending what the situation is. There are some situations where you obviously need something more potent. But even in those situations, I think there’s always a place for natural medication, either as a stand-alone treatment or in combination. This is just based on what I’ve seen, not just what I think, what I’ve seen from what people say. (D9P16-F)
9	I would think in the most part people are very reactive. I don’t know that people would proactively engage in conversations a lot in my experience. But I think if they were asked, then they would provide evidence-based information to the best of their knowledge. (D6P10-I)
	*Navigating practice in a retail environment*
10	I own a pharmacy… I still work in the shop on a daily basis. So I still come across on a daily basis having to chat to people about this. But then I am also going to come at it from the side [that complementary medicines] prop up half of the bank loan. So I guess we are going to go both ways on this a little bit. (D5P7-F)
11	If a pharmacy’s going to lose money for the sake of a sale, that isn’t a good enough reason for the sake of giving something out. We should always be having a look at evidence-based treatments,… (D4P5-I)

The code provides the discussion number, the participant number, and whether it was an interview or focus group

Other participants adopted a reactive approach to complementary medicines. These participants indicated that they are less likely to initiate discussion of complementary medicines with consumers, and were more likely to express a lack of confidence in complementary medicines. Participants expressing these views tended to rely more heavily on support staff in this area (Table 4, quote 3). For some participants, a reactive role towards complementary medicines was seen as a consequence of the lack of evidence for the effectiveness of many of these medicines (Table 4, quote 4).

#### Approach to evidence

Participants also varied in their approach to evidence. Most participants explicitly endorsed “evidence-based practice” in relation to complementary medicines, but what participants took this to mean and how it related to their day-to-day practice differed.

Many participants described their practice in a way that is consistent with evidence-based practice while also recognising some of the challenges (Table 4, quotes 5 and 6). Some participants, however, expressed views inconsistent with evidence-based practice (Table 4, quote 7 and 8). These participants put significantly more weight into anecdotal reports and placebo effects as providing evidence for the effectiveness of complementary medicines.

**Fig. 1:**
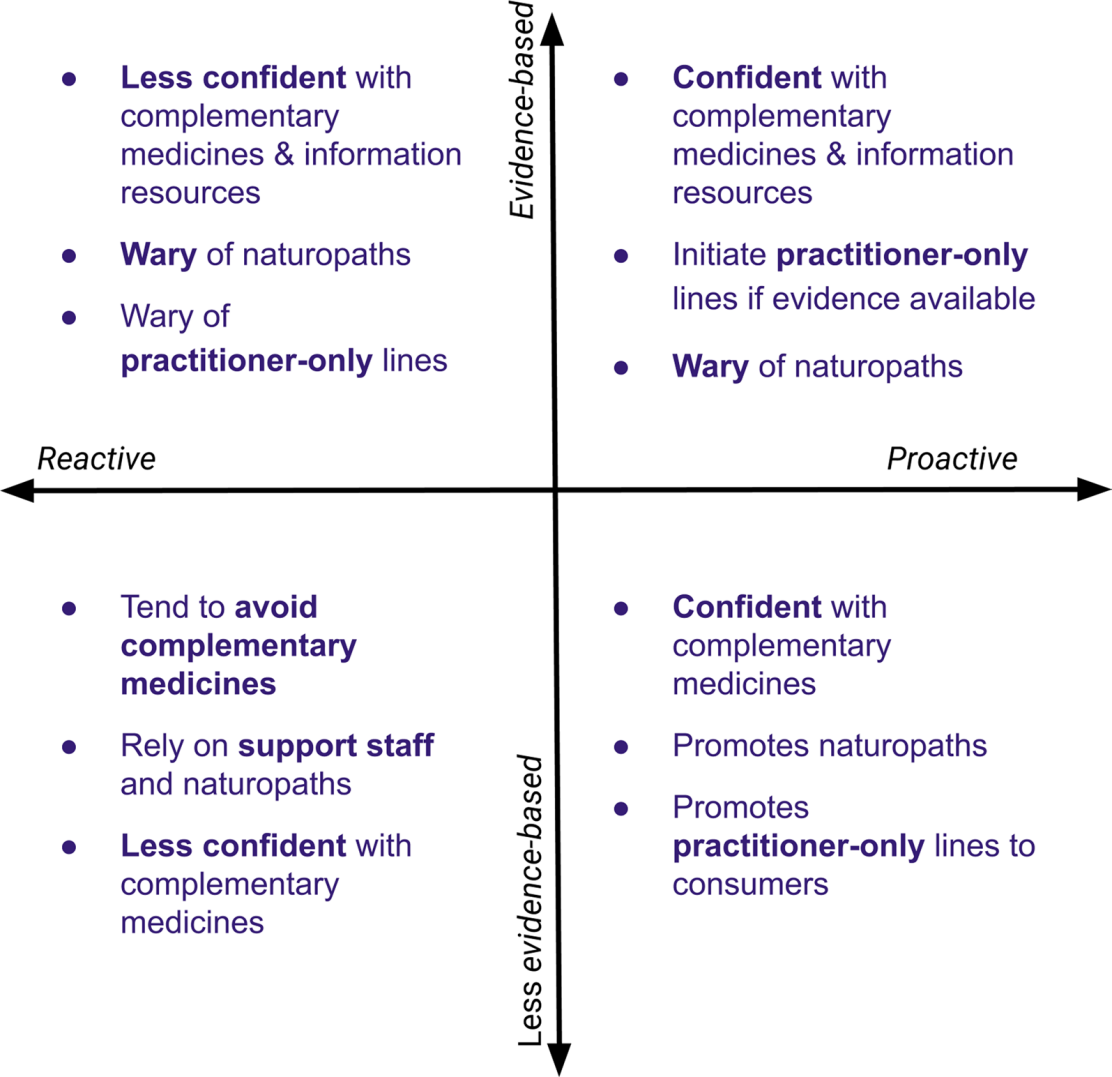
How participants varied in relation to how they viewed the context of providing advice on complementary medicines in community pharmacy

Different participants expressed each of the following views: “proactive and evidence-based”, “reactive and evidence-based”, “proactive and less evidence-based” and “reactive and less evidence-based”. Table 4, quote 9 provides an example of the participants suggesting that most pharmacists they knew were “reactive and evidence-based”. Where participants exist on these spectra informed their response to a variety of topics regarding the sale of complementary medicines in community pharmacy, including the role of naturopaths, the availability and confidence the participant had with regard to information resources, practitioner-only products and the proposed framework. How participants approached the context of complementary medicine sales in community pharmacy according to the key themes is summarised in Fig. 1.

#### Navigating practice in a retail environment

All participants discussed implications of the retail environment within the context of fulfilling their professional obligations. Participants who were pharmacy owners, in particular, recognised the impact of complementary medicines on the financial bottom-line of the pharmacy (Table 4, quote 10). Participants differed, however, in how they navigated practice in the retail environment. Most participants sought to prioritize professional obligations over financial considerations (Table 4, quote 11). These participants focused on ensuring appropriate practice within the confines of financial constraints. Because participants differed in how they viewed appropriate practice in the context of complementary medicines, they also differed on the financial impost they were willing to accept to fulfil the responsibilities outlined in the proposed framework. This topic is discussed in detail below in relation to the acceptability and feasibility of the framework.

### Acceptability of the framework

**Fig. 2:**
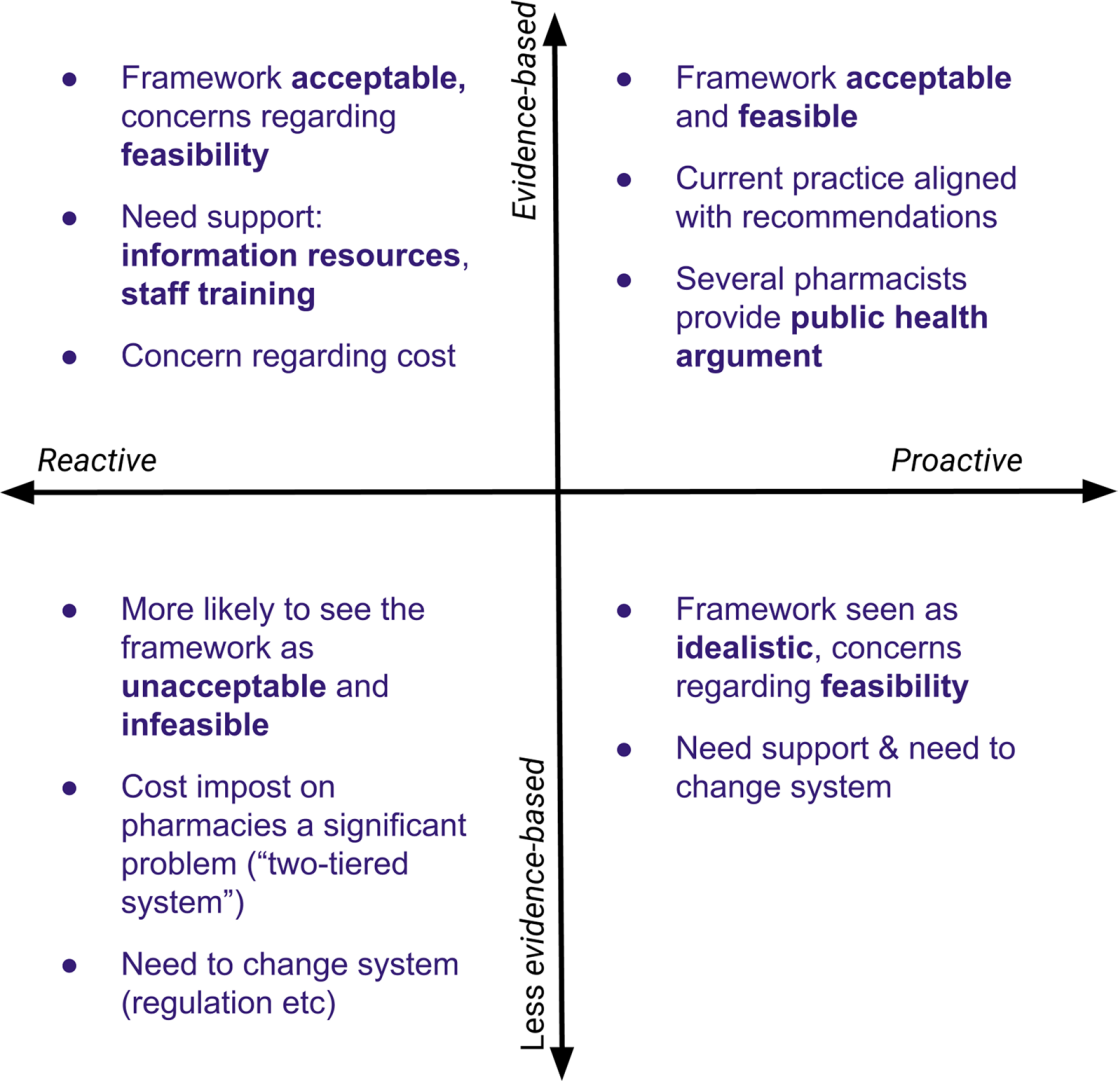
How participants views on the acceptability and feasibility of the framework different depending on how they approach complementary medicines and evidence

Most participants felt the framework was acceptable: that it captured the responsibilities of pharmacists when selling complementary medicines. Participants were more likely to express concern regarding the feasibility of the framework. Participant views on the acceptability and feasibility of the framework was informed by how they approached complementary medicines and how they approached evidence. The other key determinant was how the participant navigated practice within a retail environment. Participant views regarding the acceptability and feasibility of the framework are summarised in Fig. 2.

Participants who were “proactive” and “evidence-based” were strongly in favour of the proposed ethical framework. These participants saw the framework as being closely aligned with their practice and commended the clear guidance the framework provided regarding pharmacist responsibilities when selling complementary medicines. (Table 5, quote 1 and 2). Several participants provided an argument along the lines of the public health benefit to support the sale of complementary medicines in community pharmacies. (Table 5, quote 3 provides an example).

**Table 5:** Quotes from participants regarding the acceptability of the framework

	Participant quotes
	*The framework is acceptable and compatible with practice*
1	I think generally pharmacists are time poor and stressed and overburdened, so anything that can make something more simplified and streamlined with clear-cut expectations is useful. (D7P12-F)
2	I would say [the framework is acceptable], particularly with, yeah, treating it along the lines of an S2 or an S3 [over-the-counter products]. So front-shop staff can talk to the patients about it. If there’s any further queries, the pharmacist can be involved, but they don’t automatically have to come down and talk to them if it’s not something that there’s any questions about. (D7P11-F)
	*The public health argument*
3	I think the reason that pharmacies should sell their complementary medicine is not because there is a market. I think people, instead of going to the health food store to get their complementary medicine, they should come to the pharmacy because there is a better chance that the pharmacies can find out if there’s any interaction for people with some actual medications. But I know most of my customers. I know exactly what they are taking. If they come and someone on warfarin asks me for some complementary medication, I just quickly before going and checking their medical history, I know that that’s not the right thing to give to the person. But if that person goes to the health food store and buy it there, there is no way that they can figure it out. So I think they should be always at the pharmacy because people should think to go to pharmacy to get their complementary medicine because that way they are going to be protected and lots of trauma is going to be stopped. (D1P1-I)
	*A threat to the acceptability of the framework with a response from another participant*
4	I would say consumers mostly view [complementary medicines] as an item of commerce. You buy them like you buy bread and milk, in some instances, for some of them. So you’ve now imposed this cost on us providing evidence, but in order to do that, we have to mark the product up more. Then you’ve got this two-tiered system. (D5P7-F)
5	I think that having the degree means that… people come for a higher level of service and understanding than what they can get in the supermarket. And that’s part of what differentiates us professionally. And that’s part of why it’s still called a pharmacy and not a supermarket. I’m comfortable that I would actually be probably more comfortable practising where the TGA [Therapeutic Goods Administration] just says, “Yes, that is safe to take.” And then the pharmacist makes the clinical judgement and says, “Well, this may not be the best product for you.” I think that that’s literally our goal. (D5P9-F)

The code provides the discussion number, the participant number, and whether it was an interview or focus group

Two threats to acceptability were identified by participants. The primary perceived threat to the acceptability of the framework was that it permits a “two-tiered system” for the sale of complementary medicines. The framework identifies responsibilities for pharmacies selling complementary medicines that are not expected of other retailers. This point was raised in several focus groups and interviews. The quotes provided in Table 5 (quote 4 and 5) illustrate the back-and-forth between a participant who argues the framework is not acceptable due to the differential cost it imposes on community pharmacies and a second participant who argues that part of being a pharmacist involves such obligations.

The second threat to the acceptability of the framework is a consequence of the different approaches participants take to evidence-based practice. The proposed framework assumes a shared understanding of what is considered appropriate evidence for the efficacy of complementary medicines. Participants who expressed an approach to evidence for complementary medicines that was less evidence-based appear to hold a different view. Accepting placebo effects as sufficient evidence of the efficacy of complementary medicines and/or putting considerable weight into the anecdotal experiences of others are approaches to evidence that are incompatible with the framework. Similarly, for those who take the view that placebo effects and anecdotal reports are sufficient evidence for determining the efficacy of complementary medicine, the proposed framework will be viewed as unacceptable.

### Feasibility of the framework

Participants tended to be more concerned about the feasibility of the framework as opposed to its acceptability. The specific barriers that participants identified, and the kinds of things that would enable participants to overcome the barrier, depended on how participants approached complementary medicines and evidence.

Participants who were “evidence-based” and “proactive” tended to see the framework as both acceptable and feasible. Participants who were “evidence based” and “reactive” expressed concerns about the feasibility of implementing the framework. These participants tended to identify local, practical barriers and to identify areas of support that would remedy these concerns. The two most consistently identified barriers were the availability of (and confidence with) evidence-based information resources on complementary medicines and staff training. Some example quotes highlighting the importance of information resources and training and some of the optimism participants expressed to addressing these barriers are provided in Table 6, quotes 1–3. The opportunities that might arise from addressing some these barriers was also recognised (Table 6 quote 4). A number of participants identified an increased focus on practitioner-only lines as one way to differentiate pharmacy services in relation to complementary medicines while fulfilling the professional obligations outlined in the framework (Table 6 quote 5).

**Table 6:** Quotes from participants regarding the feasibility of the framework

	Participant quotes
	*Practical local barriers need to be addressed*
1	Oh. I think it’s a very nice framework in an ideal world, and if we are provided with tools and training and the resources to train the staff, I would be very happy to have that in the pharmacy. (D2P2-F)
2	I feel like it would be really helpful if there was a better database to look up interactions and all that type of thing because more often than not, I have to call either the company or look into it really far to make sure it doesn’t interact. So maybe extra training in that area like compulsory training, I guess. (D3P4-I)
3	So I think, honestly, I would just be keen to try it out in the shop and see how it actually works. But it’s sort of one of those questions. If you change the framework and require pharmacies to do something, some sort of fundamental change in how we provide advice, would that open up the space for a new database to actually provide some money so someone would actually make it? Would that then mean that companies looking to get their products into pharmacy would put more emphasis on evidence and therefore training? So pharmacists wouldn’t have to be doing these trainings. You’re going to get detailed by companies that are looking to get the best, most evidence-based product into your stores. (D5P9-F)
	*Additional training is needed and this is an opportunity*
4	I’ve got 30 staff, and the idea that there could be more specialised training for people that have that interest [in evidence-based complementary medicines] and that could be another avenue for non-pharmacists into pharmacy careers. Immediately, that’s more attractive than going to work at Woolies [a large supermarket chain], where they just sell the stuff *en mass* for profit. How would that not be a good thing when we’ve copped a lot of bad press about some pharmacists? So yeah, definitely. I would be very interested to see if this framework allowed for more of that. (D5P9-F)
	*The potential role of practitioner-only lines*
5	Well, I feel like there should be, I guess, a shift away from the front-shop selling. So just to distinguish pharmacy from the health food store, so other things that people just see on TV or things that people can buy without talking to a pharmacist or talking to someone that’s been trained in complementary medicines. So there’s the idea of what we’ve got, the pharmacy, with labelling it, even though it’s not necessarily a dangerous product, but just something that at least requires a consult from the first go. Not every time but just from the initial, first-selling to them so they know exactly why they’re taking it, rather than just they’ve been taking it for 10 years. And if we said, “Well, this is a better product, a better form of calcium or whatever it might be,” at least that way they can think of their complementary medicines along the same lines as their regular medications. So they still put some value on it, and they don’t just look for the cheapest option or the most convenient, necessarily, but something that they get more value out of. (D7P11-F)
	*Large-scale system change is needed*
6	…[G]etting all the pharmacies on the same page. If you’ve got pharmacies that are run by corporations and banner groups that are more for-profit versus small community pharmacies that are trying to provide a service. You’ve got to have these frameworks that are enforceable, maybe through QCPP [Quality Care Pharmacy Program] or a PBS [Pharmaceutical Benefits Scheme] listing, and make sure that everyone does the same things and stocks the same products and doesn’t stock the same products based on evidence. (D7P12-F)
7	The next thing, I think, would be TGA [Therapeutic Goods Administration]. If they’re approving it, but then it’s not evidence based, then consumers will get confused because they would say, “Oh, but then it’s approved by TGA, so it must be all right or evidence based.” (D10P17-I)
8	Why should it be up to us as pharmacists? Why shouldn’t the TGA—when it goes to them in the first place to be approved, why is it even getting to us? Why are we required to make the decision? Why haven’t TGA done their job? (D5P7-F)

The code provides the discussion number, the participant number, and whether it was an interview or focus group

Participants who described their practice in a way that was less evidence-based tended to agree with the barriers and facilitators identified above as well as express additional concerns regarding the feasibility of implementing the framework. Participants who were “proactive” and “less evidence-based” tended to see the framework as acceptable though idealistic, and suggest that it could only be implemented if there were significant system changes made to support the framework. Participants who were “reactive” and “less evidence-based” were more likely to view the framework as both unacceptable and infeasible. These participants had significant concerns about cost implications. The system changes that each of these groups of participants suggested were similar. Suggestions included ensuring all pharmacies implemented the framework in a similar way (perhaps taking a regulatory approach to assessing compliance with the framework) and making changes to the way the complementary medicines were regulated such that there were tighter restrictions on the availability of complementary medicines or labelling requirements to indicate the strength of evidence for an effect. (Table 6, quotes 6–8 provide examples).

The key topics discussed in interviews were similar to those discussed by individuals within focus groups. Differences in perspectives between participants working in different locations and different types of pharmacy were not observed.

## Discussion

The proposed ethical framework provides specific guidance to pharmacists on fulfilling their responsibilities when selling complementary medicines. The framework seeks to address current gaps in the professional and academic literature. It addresses apparent conflicts in pharmacist responsibilities for promoting positive health outcomes and respecting consumer health beliefs while recognising the current regulation of these products and the roles that pharmacists play in providing advice on complementary medicines. The degree to which the framework can be implemented relies on several factors, important among these is whether community pharmacists find the framework acceptable and feasible. The community pharmacists participating in the study differ in how proactive they were in relation to complementary medicines, the degree to which they adopted an evidence-based approach to their practice, and how they navigated practice in a retail environment. These factors informed the way in which participants approached the sale of complementary medicines in practice and how they approached the proposed framework. Most pharmacists in this study found the proposed framework to be acceptable and feasible to implement with targeted support.

The barriers and challenges to accepting additional responsibilities in relation to complementary medicines identified by participants in this study resemble those identified in recent literature [16, 20, 23]. These include limited access to unbiased information, a perceived lack of education and financial disincentives to being more proactive in the provision of advice [23]. The suggested solutions to addressing the barriers are also similar: education and training, improved workplace resources and support and a range of broader system-level changes (such as improving the evidence-base and regulatory changes) [16, 20]. The key contribution of the present study is to explicitly address the lack of clarity regarding the roles and responsibilities of pharmacists that has been identified [16, 23], and then seek to explore how participants respond to the specific responsibilities proposed. This provided a way to identify the key characteristics that informed how pharmacists responded to these responsibilities and how individual pharmacists differed in terms of their response.

With the right support participants who are evidence-based and proactive towards complementary medicines might act as champions for the framework. To adequately support these pharmacists it will be important to address the barriers identified by participants who felt the framework is acceptable but had concerns regarding feasibility. Appropriately addressing these barriers should help to shift more pharmacists towards an evidence-based and proactive approach to complementary medicines. Several participants indicated the need for better access to and confidence with evidence-based information resources on complementary medicines. The provision of guidance on the availability, strengths and limitations of key evidence-based information resources available to community pharmacists could aid pharmacists unfamiliar with these resources. A second and related activity is the availability of high-quality training for pharmacists and pharmacy support staff. There is a lot of sponsor-provided training opportunities in community pharmacy for both pharmacists and pharmacy support staff. Participants in this study identified the need for independent evidence-based learning opportunities in complementary medicines for both pharmacists and pharmacy support staff. Similar needs have been identified in the literature [4, 17, 23]. Learning activities specifically targeting evidence-based health care and the role of pharmacists and pharmacy support staff in providing appropriate advice regarding complementary medicines should facilitate implementation of the framework. These learning activities could then feed into longer-term projects such as the development of a career pathway for pharmacy support staff with credentials and expertise in providing evidence-based advice regarding complementary medicines. These activities build on the recognition of the roles and responsibilities of pharmacy support staff in providing health information within community pharmacy, and the increasing opportunities for training and support for advanced roles [33–35].

The initiatives outlined above are targeted at pharmacists who identify an evidence-based approach to complementary medicines but experience barriers to taking a more proactive approach to complementary medicines. We suggest that focusing on these initiatives is likely to be an effective and efficient strategy to improve pharmacy practice in relation to complementary medicines. The framework clarifies pharmacist responsibilities, while the additional training activities and further guidance on evidence-based information resources would build the capacity of pharmacists and pharmacy support staff to deliver evidence-based advice to consumers using complementary medicines. There are also larger-scale regulatory and system changes that can further support pharmacists and the public in relation to complementary medicines. While it is important to discuss, debate and progress these system changes, these larger-scale items were not impediments to implementing practice change for most participants.

The strengths of the study include asking participants to respond to specific advice on the responsibilities of pharmacists when selling complementary medicines, the variation present in the demographic details of the participants, and the in-depth examination of how the participants approached complementary medicines and the framework. Key limitations include the small size of the study and the possibility that pharmacists with particular views were more likely to participate or not participate in the study. The recruitment methods relied heavily on social media and participation was online, both of which may have discouraged some pharmacists from participating. This initial work could be used to inform a more comprehensive evaluation to determine whether the themes identified in this study can be identified and measured in a larger representative sample of community pharmacists. While many of the issues discussed are relevant to pharmacists practising internationally, further work is needed to explore the extent to which these findings apply outside of the Australian context. It is also important to extend this work to include the perspectives of key stakeholders to the proposed framework. This would include consumers, pharmacy support staff, regulators, leaders of peak professional groups and the complementary medicines industry. This work would help to facilitate the incorporation of the ethical framework into formal professional guidance and standards.

## Conclusion

The *Framework for pharmacist responsibilities when selling complementary medicines* addresses an important gap in providing specific professional guidance to pharmacists when selling complementary medicines. The results of the study suggest that the framework may be acceptable to community pharmacists and be feasible to implement with targeted support.

## Supplementary information

**Additional file 1:** A framework for pharmacist responsibilities when selling complementary medicines. This document provides an overview of the framework that was provided to participants.

**Additional file 2:** Discussion prompts. The discussion prompts used to facilitate discussion in the focus groups and interviews.

## Data Availability

The data that support the findings of this study are available on request from the corresponding author, AL. The data are not publicly available due to the nature of qualitative research and protections on participant privacy.
