# Supplementary material for: Improving pharmacy practice in relation to complementary medicines: a qualitative study evaluating the acceptability and feasibility of a new ethical framework in Australia
Source: BMC Med Ethics. 2021 Jan 6;22:3. doi: 10.1186/s12910-020-00570-7 (PMC7788988; doi:10.1186/s12910-020-00570-7)
Supplement: Supplementary file 1 — Additional file 1: A framework for pharmacist responsibilities when selling complementary medicines. This document provides an overview of the framework that was provided to participants. [file 12910_2020_570_MOESM1_ESM.pdf]

A framework for pharmacist responsibilities when selling complementary medicines

Amber Salman Popattia
Adam La Caze

24 July 2019

Contents

Synopsis 1
Background 1
Current Professional Guidance 2
The Framework 2
Bibliography 6

Synopsis

There has been limited discussion regarding the responsibilities of pharmacists when selling complementary medicines. The proposed framework seeks to address this gap by providing guidance regarding the professional responsibilities of pharmacists when selling complementary medicines. The key responsibilities of pharmacists when selling complementary medicines include: provide recommendations consistent with best evidence; provide sufficient information to the consumer so that they can make an informed choice; intervene if a consumer is at risk of harm from complementary medicine use; and, conduct training and set up processes within the pharmacy such that all staff involved in selling complementary medicines provide appropriate advice.

Background

Many consumers use complementary medicines and choose to purchase these complementary medicines from community pharmacies.[1] Recent reports have questioned the role of pharmacists in selling and recommending complementary medicines when many lack of evidence for effectiveness.[2–6] These reports raise a number of questions. Should pharmacists sell complementary medicines if they lack rigourous evidence that they work? If pharmacists choose to sell complementary medicines, what are their responsibilities when they do?

Responses to the first question fall into two camps. Those that argue that as health professionals pharmacists should not sell any treatment that lacks evidence of effectiveness.

And those that argue that the current practice of selling complementary medicines in pharmacies is appropriate. Usually this argument is supported in terms of meeting consumer needs.

The question regarding pharmacist responsibilities when selling complementary medicines is frequently neglected. The framework provided below seeks to address this gap. We think it is important to address this question because:

1. Complementary medicines *are* frequently sold in community pharmacies
2. Consumers frequently seek advice from pharmacists regarding safe use of complementary medicines, and
3. Being clearer about the responsibilities of pharmacists when selling complementary medicines helps to resolve the question of whether it is appropriate for pharmacists to sell complementary medicines

Current Professional Guidance

Professional guidance regarding pharmacists selling complementary medicines has tended to focus on (i) respecting consumer choice, and (ii) ensuring that pharmacists' practice according to best evidence.[7] This general guidance leaves it up to the pharmacist to determine their responsibilities when selling and recommending complementary medicines.

In the past 12–18 months advice from the Pharmaceutical Society of Australia has become more explicit. In addition to providing the standard general advice, the current Pharmaceutical Society of Australia's *Code of Ethics for Pharmacists* provides the following directive:[8]

> (Pharmacists) will only purchase, supply or promote any medicine, complementary medicine, herbal remedy or other healthcare product where there is credible evidence of efficacy and the benefit of use outweighs the risk. (Integrity principle 1(h))

This advice is repeated in the Pharmaceutical Society of Australia's position statement on complementary medicines.[9]

The explicit advice provided by the Pharmaceutical Society of Australia differs from current practice regarding complementary medicines and comes without a detailed discussion outlining the position.

The Framework

If pharmacists choose to sell complementary medicines, what are their responsibilities when they do? Answering this question allows us better address the question of whether it is appropriate for pharmacists to sell complementary medicines. It is appropriate for pharmacists to sell complementary medicines to the extent that they meet their responsibilities (as outlined by the framework).

The framework starts by recognising the following points:

- Complementary medicines are regulated in such a way that they are available for consumers to purchase from a wide range of outlets.
- Despite being considered sufficiently safe for self-care, the use of complementary medicines can cause harm through adverse reactions, drug interactions and if consumers delay or forego treatments recommended by health professionals.
- Pharmacists are accessible and have the knowledge and skills to support consumers using complementary medicines safely.
- Pharmacists have overall responsibility for pharmacy processes regarding the sale of complementary medicines, staff training and the advice provided pharmacy support staff.

The focus of the framework is to outline the responsibilities pharmacists need to meet to ensure that the sale of complementary medicines supports consumers using complementary medicines safely and minimizes inappropriate use. The basis for the framework rests in principle-based ethics.[10] The responsibilities have been derived through a process of weighing up specific principles, including respect for consumer choice and the need for health professionals to promote beneficial consumer outcomes and practice according scientific evidence.

The framework proposes the following five responsibilities when selling complementary medicines:

1. Pharmacists should provide evidence-based recommendations to the consumers regarding complementary medicines
2. Pharmacists should train all staff in a pharmacy and ensure that they provide evidence-based recommendations regarding complementary medicines and seek advice of a pharmacist when required
3. When providing advice, pharmacists should provide sufficient information for consumers to make informed decisions regarding complementary medicines
4. Pharmacists should setup the pharmacy so that consumers are provided an offer of advice from a pharmacist and pharmacists are available to provide that advice
5. Pharmacists must be vigilant for complementary medicine harm and intervene if risk of harm is significant

Further details regarding these responsibilities are provided below. The responsibilities of the pharmacist in a specific context depends on what the consumer requests (see Figure 1).

Pharmacists should provide evidence-based recommendations to the consumers regarding complementary medicines

Pharmacists need to assess the effectiveness of complementary medicines based on the available scientific evidence. Any recommendation for a consumer to take a complementary medicine needs to be justifiable on the basis of this evidence.

'Scientific evidence' in this context is consistent with the advice of evidence-based medicine. Where available, randomized trials are the best evidence for assessing the effectiveness of

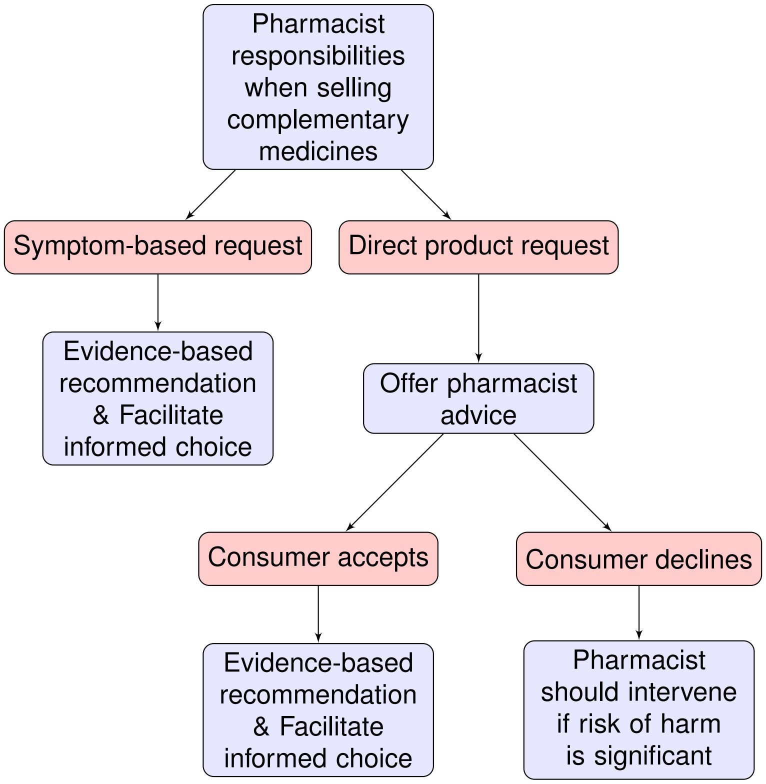

Figure 1: Flowchart for pharmacist responsibilities when selling complementary medicines

complementary medicines. When randomized trials are not available, health professionals need to make decisions based on the best outcome evidence available and an understanding of the mechanisms by which complementary medicines may benefit or cause harm.

Anecdotal evidence and the expectation of placebo effects are not a sufficient basis for recommending complementary medicines on the basis of effectiveness.

Pharmacists should train all staff in a pharmacy and ensure that they provide evidence-based recommendations regarding complementary medicines and seek advice of a pharmacist when required

The pharmacist-in-charge in a pharmacy has overall responsibility for all the advice provided in the pharmacy. The pharmacist-in-charge is responsible for ensuring all staff make recommendations that are consistent with the evidence.

Pharmacy owners and managers are responsible for training staff to ensure that all recommendations made in a pharmacy about complementary medicines are consistent with the evidence and that staff are trained to seek the advice of a pharmacist when required.

When providing advice, pharmacists should provide sufficient information for consumers to make informed decisions regarding complementary medicines

Providing information to consumers regarding complementary medicines is important to ensure that consumers are in a position to make an informed decision.

Pharmacists should provide information that is based on the existing scientific evidence regarding complementary medicines. This includes informing consumers about the benefits and risks of specific complementary medicines, the availability (or absence) of evidence supporting the use of the complementary medicine, and alternative options if better evidence exists for the benefits of these options.

Pharmacists should setup the pharmacy so that consumers are provided an offer of advice from a pharmacist and pharmacists are available to provide that advice

Pharmacists should put in place processes that ensure all consumers making direct requests for complementary medicine product(s) are provided an offer of pharmacist advice.

If a consumer accepts the offer for advice, pharmacists have a responsibility to ensure that they are available to provide evidence-based advice to the consumers regarding complementary medicines.

Pharmacists must be vigilant for complementary medicine harm and intervene if risk of harm is significant

When giving advice about and/or recommending complementary medicines, pharmacists need to assess the possible risks associated with the use of the complementary medicine and work with the consumer to minimise the risk of harm.

Ensuring that consumers purchasing complementary medicine are offered the opportunity for further advice from the pharmacist is an important mechanism to minimise the risk of harm from complementary medicines.

When consumers elect to purchase complementary medicines without further advice from the pharmacist, it can be harder to assess risk of harm. Nonetheless, pharmacists still have responsibilities to ensure pharmacy staff are adequately trained to identify and respond to situations in which consumers may be at risk of harm (for example, if the consumer is buying excess quantities of a complementary medicine). Pharmacists also have a responsibility to intervene if they observe purchases of complementary medicines that may lead to harm, even if the consumer has not specifically requested further advice from the pharmacist.

Bibliography

1. Williamson, M., Tudball, J., Toms, M., Garden, F. & Grunseit, A. *Information Use and Needs of Complementary Medicines Users*. (2008).

2. Bray, K. Is your pharmacist giving you the right advice? (2017). Available at: https://www.choice.com.au/health-and-body/health-practitioners/doctors/articles/pharmacy-advice-for-stress.

3. Thompson, G., Russell, A. & Fallon, M. SWALLOWING IT. (2017). Available at: http://www.abc.net.au/4corners/stories/2017/02/13/4616948.htm.

4. Arnold, A. Should you trust your pharmacist? (2016). Available at: http://www.abc.net.au/radionational/programs/backgroundbriefing/should-you-trust-your-pharmacist/7237496.

5. King, S. *Review of Pharmacy Renumeration and Regulation: Final Report*. (2017).

6. Mannix, L. Shops full of vitamins, miracle pills 'trashing pharmacists' reputation'. (2019). Available at: https://www.smh.com.au/national/shops-full-of-vitamins-miracle-pills-trashing-pharmacists-reputation-20190621-p5201i.html. (Accessed: 2nd July 2019)

7. Pharmacy Board of Australia. *Code of conduct*. (AHPRA, 2014).

8. The Pharmaceutical Society of Australia. *Code of ethics for pharmacists*. 52 (2017).

9. The Pharmaceutical Society of Australia. *Complementary Medicines: Position Statement*. (2018).

10. Beauchamp, T. L. & Childress, J. F. *Principles of Biomedical Ethics*. (Oxford University Press, 2012).
