# Supplementary material for: Improving pharmacy practice in relation to complementary medicines: a qualitative study evaluating the acceptability and feasibility of a new ethical framework in Australia
Source: BMC Med Ethics. 2021 Jan 6;22:3. doi: 10.1186/s12910-020-00570-7 (PMC7788988; doi:10.1186/s12910-020-00570-7)
Supplement: Supplementary file 2 — Additional file 2: Discussion prompts. The discussion prompts used to facilitate discussion in the focus groups and interviews. [file 12910_2020_570_MOESM2_ESM.pdf]

Workshop introduction and discussion prompts

Adam La Caze Amber Salman Popattia Laetitia Hattingh

Introduction

Welcome. Thank you for participating in this project.

The aim of the project is to assess the acceptability and feasibility of an ethical framework for determining pharmacist responsibilities when selling complementary medicines. The workshop will last approximately 60 minutes and assumes you have read the pre-workshop material and completed the survey.

People hold different views regarding the responsibilities of pharmacists when selling complementary. The purpose of this project is to discuss and reflect on your views and the views of others. There are no predetermined “right” or “wrong” answers to the topics under discussion. An important aim of the workshop is to seek to understand the different views that pharmacists hold on the topic of complementary medicines, and specifically the proposed framework. We thank you for sharing your views and welcome different opinions.

The information you provide will remain confidential. The workshop will be video and audio recorded. All identifying information will be stored securely and only be available to the researchers. All participants will de-identified in the transcripts made of the workshop. A short summary of the key themes discussed in the workshop will be emailed to you and you will have an opportunity to comment on the summary.

We ask you to use your first name for the purposes of the workshop, and we will discuss location of practice during the workshop. Please refrain from providing further identifying information where possible (e.g. specific names of pharmacies or pharmacists). Please also treat all information discussed within the workshop regarding fellow participants as strictly confidential.

Participant introductions

We will ask all participants to introduce themselves and briefly describe the work they do in community pharmacy (including location in broad terms, the type of pharmacy they work in and their current role).

Discussion prompts

1. Topic 1: Current approach to complementary medicines (and professional advice regarding complementary medicines)

i. Please describe your current involvement in stocking, recommending and selling complementary medicines? [Examples]
ii. What roles do you think pharmacists currently have in relation to selling complementary medicines? [Consumer expectations; Professional expectations; Pharmacy expectations; Pharmacist cf pharmacy support staff; Business roles?]
iii. What role do you think pharmacists *should* have in relation to selling complementary medicines? [Explore is-ought differences]
iv. Are there any challenges in pharmacists fulfilling their role(s) in relation to complementary medicines? [Lack of evidence? What do you consider as appropriate evidence for complementary medicines?]
v. What do you think about the current professional guidance regarding complementary medicines? PSA guidance (integrity principle 1(h))? Media reports? [Provide details as required]

2. Topic 2: Group responses to the proposed ethical framework. Is it acceptable?

   i. Do you think the proposed framework captures pharmacist responsibilities when selling complementary medicines? [What does it get right? What does it get wrong? What is it missing?]
   ii. Do you think other pharmacists would find the framework acceptable?
   iii. What changes would you recommend?

   Further prompts for discussion related to Topic 2.

   i. Do you think pharmacists have different responsibilities when recommending as opposed to selling complementary medicines?
   ii. Do you think pharmacists should *recommend* complementary medicines that lack clinical evidence of effect and a clear scientific rationale for producing an effect?
   iii. What responsibilities do pharmacists have to consumers purchasing complementary medicines from the pharmacy when the pharmacist isn't involved in the sale?

3. Topic 3: Feasibility and barriers to implementation. This topic assumes that the framework is acceptable to most members of the group, or that modifications to the framework could make it acceptable.

   i. Do you think you could implement the framework in your practice?
   ii. What barriers are there to implementing the framework?
   iii. What support do you think would be necessary in order to implement the framework?
